# Genome and transcriptome wide association study identify candidate genes regulating folate levels in maize

**DOI:** 10.3389/fpls.2025.1606220

**Published:** 2025-06-19

**Authors:** Chenglin Zou, Meng Yang, Aihua Huang, Runxiu Mo, Ruining Zhai, Kaijian Huang

**Affiliations:** Maize Research Institute, Guangxi Academy of Agricultural Sciences, Nanning, China

**Keywords:** TWAS, GWAS, folate, maize, candidate genes

## Abstract

**Background:**

Maize (*Zea mays* L.) serves as a crucial dietary source of folate for humans. However, the genetic regulatory mechanisms underlying the natural variation of folate in maize remain poorly understood. Here, we integrated multi-omics approaches to elucidate the molecular mechanisms governing folate accumulation in maize.

**Methods:**

A total of 380 maize kernels representing 190 maize inbred lines from China, Thailand, Mexico, and Peru were collected. RNA-seq was conducted on 380 maize kernel samples, and folate content was quantified using high-performance liquid chromatography (HPLC). The samples were stratified into high and low folate groups based on median folate values. Differentially expressed genes (DEGs) were identified between the two groups identified. Candidate genes associated with folate accumulation were further located by integrating transcriptome-wide association studies (TWAS) and genome-wide association studies (GWAS) analyses. Finally, quantitative real-time PCR (qRT-PCR) was employed to validate the expression patterns of these candidate genes.

**Results:**

A total of 137 DEGs that exhibited significant differences between the high-folate and low-folate groups were identified. Gene Ontology (GO) and Kyoto Encyclopedia of Genes and Genomes (KEGG) enrichment analyses revealed that these genes were significantly enriched in pathways related to phenylpropanoid biosynthesis, oxidoreductase activity, and stress response. GWAS identified 2,153 candidate genes associated with folate traits (*P* ≤ 1.00E-05). Through the integration of DEGs and the intersection of genes identified by GWAS, seven candidate genes were further identified. In addition, TWAS analysis further identified 13 causal genes associated with the candidate genes, which are involved in folate biosynthesis. In addition, the expression levels of these candidate genes were validated by qRT-PCR experiments, suggesting significantly higher expressions in the high folate group compared to the low-folate group.

**Conclusion:**

This study identifies key regulatory genes potentially influencing folate accumulation in maize and provides critical insights for the development of biofortified maize varieties with enhanced nutritional value.

## Introduction

Maize is one of the most important food crops globally, playing a pivotal role in global food production (Guo et al., 2019). Maize is not only a crucial component of the human diet but also contains a wealth of nutrients. Folate represents an essential micronutrient that plays critical roles in human health maintenance and disease prevention (Shulpekova et al., 2021). This water-soluble vitamin serves as a key cofactor in fundamental biological processes including DNA synthesis and repair, amino acid interconversion, and cellular proliferation through its involvement in one-carbon metabolism (Su et al., 2024). However, humans are incapable of endogenous folate biosynthesis due to the absence of key enzymatic pathways, making dietary intake the sole source of this vital nutrient. Despite its importance, the folate content in maize grains is generally low (Blancquaert et al., 2015). Therefore, investigating the molecular mechanisms to enhance folate content in maize not only elevates its nutritional value but also holds great significance for optimizing human dietary structures and improving the quality of life.

Recent years have witnessed substantial advancements in elucidating the molecular regulatory mechanisms governing folate accumulation, driven by the application of genomic sequencing technologies and marker-assisted selection strategies (Pan et al., 2024; Xia et al., 2023). Emerging evidence suggests that folate biosynthesis during late-stage maize kernel development may be coordinately regulated through interrelated metabolic pathways, including pyruvate metabolism and glutamate metabolism (Lian et al., 2022). This discovery of multi-pathway regulatory interactions provides novel insights into the complex regulatory networks underlying folate biosynthesis in cereal crops. Recent investigations have demonstrated significant positive correlations between folate accumulation and the expression levels of *SiADCL1* and *SiGGH* genes during millet panicle development, suggesting their pivotal role in modulating folate metabolic flux (Hou et al., 2022). Parallel transcriptomic analyses in maize endosperm have systematically mapped gene networks governing folate biosynthesis, identifying critical gene modules and putative regulatory elements (Song et al., 2021). GWAS have emerged as a powerful tool for detecting genetic variants linked to complex traits, with a notable study revealing 95 loci significantly associated with grain folate content, including a key gene encoding 5-formyltetrahydrofolate cycloligase that directly influences folate derivatives (Xiao et al., 2022). While GWAS effectively pinpoints trait-associated SNP loci, technical limitations persist as these variants often reside in non-coding regions with ambiguous functional annotations (Visscher et al., 2017). TWAS complement GWAS by establishing direct links between gene expression patterns and phenotypic variations at single-gene resolution, a methodological advancement particularly valuable for plant genome research (Gusev et al., 2016; Wainberg et al., 2019). A pioneering study integrating GWAS and TWAS across 421 soybean accessions successfully deciphered the genetic architecture of seed weight and oil content, uncovering coordinated molecular networks regulating these agronomic traits (Yuan et al., 2024). Despite these methodological advances, the systematic integration of multi-omics approaches to elucidate folate regulatory networks in maize—particularly through combined genomic and transcriptomic analyses—remains underexplored, highlighting a critical knowledge gap in cereal biofortification research.

In this study, 380 kernel samples derived from 190 maize inbred lines were collected and assessed for folate content, with samples subsequently categorized into high-folate and low-folate groups. By utilizing a combination of transcriptomic analyses, GWAS, and TWAS, candidate genes associated with folate content in maize kernels were identified. The study also elucidated the functions and regulatory roles of these genes in maize folate metabolism. These findings provide a theoretical foundation for future efforts to enhance folate content in maize through molecular breeding.

## Materials and methods

### Plant materials and folate assay

In this study, 380 maize grain samples representing 190 different maize inbred lines were selected as experimental materials. These samples encompass a wide range of genetic backgrounds and originate from China, Thailand, Mexico, and Peru, among other locations. For each maize inbred line, two samples were selected, each consisting of 5 kernels for grinding and subsequent folate determination using the HPLC method. Based on the median folate content, the maize inbred lines were categorized into high-folate and low-folate groups. The sample details have been provided in **Supplementary Table S1**.

### RNA-seq and data analysis

The total RNA was extracted using the RNAprep Pure Plant Kit (Tiangen, Beijing, China) according the instructions provided by the manufacturer. The constructed libraries were sequenced on the Illumina Novaseq 6000 sequencing platform. The raw reads were further processed with a bioinformatic pipeline tool, BMKCloud (www.biocloud.net) online platform. To ensure the accuracy, reads with more than 10% N bases and low-quality reads with Q ≤ 10 and over 50% bases were excluded. After filtering low-quality reads, these clean reads were then mapped to the reference genome sequence (B73 RefGen_v3) using Hisat2. Only reads with a perfect match or one mismatch were further analyzed and annotated based on the reference genome.

### Differential gene expression and functional enrichment analysis

Differential expression analysis of two groups was performed using the DESeq2. Gene expression levels were calculated using fragments per kilo base of transcript per million mapped reads (FPKM). The resulting *P* values were adjusted using the Benjamini and Hochberg’s approach for controlling the false discovery rate. Genes with an adjusted *P*-value < 0.01 & Fold Change ≥ 2 found by DESeq2 were assigned as DEGs. GO enrichment analysis was implemented by the clusterProfiler packages based Wallenius non-central hyper-geometric distribution (Young et al., 2010). The clusterProfiler software were used to test the statistical enrichment of differential expression genes in KEGG pathways (Yu et al., 2012). Gene set enrichment analysis (GSEA) was performed with GSEA v3.0 (http://www.broadinstitute.org/gsea/).

### Population genetics analyses

To analyze the phylogenetic relationships of the accessions, we constructed an unrooted/rooted phylogenetic tree using the neighbor-joining method with the Kimura 2-parameter/p-distance model in MEGA-CC software (MEGAX) (Kumar et al., 2018), with 1000 bootstrap replicates. In total, 403,933 high-confidence SNPs were used to infer the population structure within accessions using ADMIXTURE (v1.22) (Alexander et al., 2009), K values (the putative number of populations) ranging from 1 to 10. We assessed the number of sub-populations using five-fold cross-validation. The Q matrix for each K value stacked assignment bar plots were generated using the R package Pophelper (http://royfrancis.github.io/pophelper). Principal component analysis (PCA) of the SNPs was performed using smartPCA program from the EIGENSOFT package using the default parameters (Price et al., 2006).

### Variants calling and GWAS analysis

The SNPs markers were identified using the transcriptome data. Briefly, BAM files generated after the mapping process with HISAT2 were sorted using SAMtools (v0.1.19). Next, duplicate reads were removed with Picard tools (v2.25.7) (https://github.com/broadinstitute/picard). Finally, GATK (v3.8) was employed to call variants using the parameters “–indelSizeToEliminateInRefModel 50 –variant_index_type LINEAR –sample_ploidy 2 -nct 4 -U ALLOW_N_CIGAR_READS”. SNPs with a minor allele frequency (MAF) less than 0.01 or an integrity threshold below 0.5 were filtered out. Finally, 403,933 SNPs were retained for subsequent analysis. GWAS was performed using a Linear Mixed Model (LMM) in the GEMMA (V0.98.1) package (Zhou and Stephens, 2012). The matrix of pairwise genetic distances calculated by GEMMA and the PCA calculated by smartPCA were used as the variance-covariance matrix of random effects. The Manhattan and quantile quantile (QQ) plots of GWAS results were generated in R software (https://cran.r-project.org/).

### TWAS analysis

To determine the relationship among candidate transcripts, eQTL analysis was performed by TRAS of 190 maize inbred lines using the LMM model. In our eQTL analysis, the 403,933 SNPs were defined as markers, and the expression of candidate transcripts were considered as phenotypes. To avoid potential false positives in multiple comparisons, the modified Bonferroni correction was used to determine the genome-wide significance thresholds of the TWAS, the more rigorous criterion of α=0.01/number of markers was used and the candidate genes were determined based on a threshold of *P* < 2.476e-08.

### qRT-PCR

Total RNA was extracted from maize kernels using the FlaPure Plant Total RNA Extraction Kit following the manufacturer’s protocol. Subsequently, after DNase treatment, Reverse transcription was performed using the UnionScript First-strand cDNA Synthesis Mix. The resulting cDNA was then utilized as a template for quantitative PCR, which was performed with the GS AntiQ qPCR SYBR Green Fast Mix on the CFX96 Touch Real-Time PCR Detection System, including three biological replicates. The gene expression levels were normalized to the expression of theβ-actin and calculated for each sample using the 2^−ΔΔCT^ method. The information of the primers is shown in **Supplementary Table S2**.

## Results

### Genetic diversity and folate content analysis in maize

**Figure 1:**
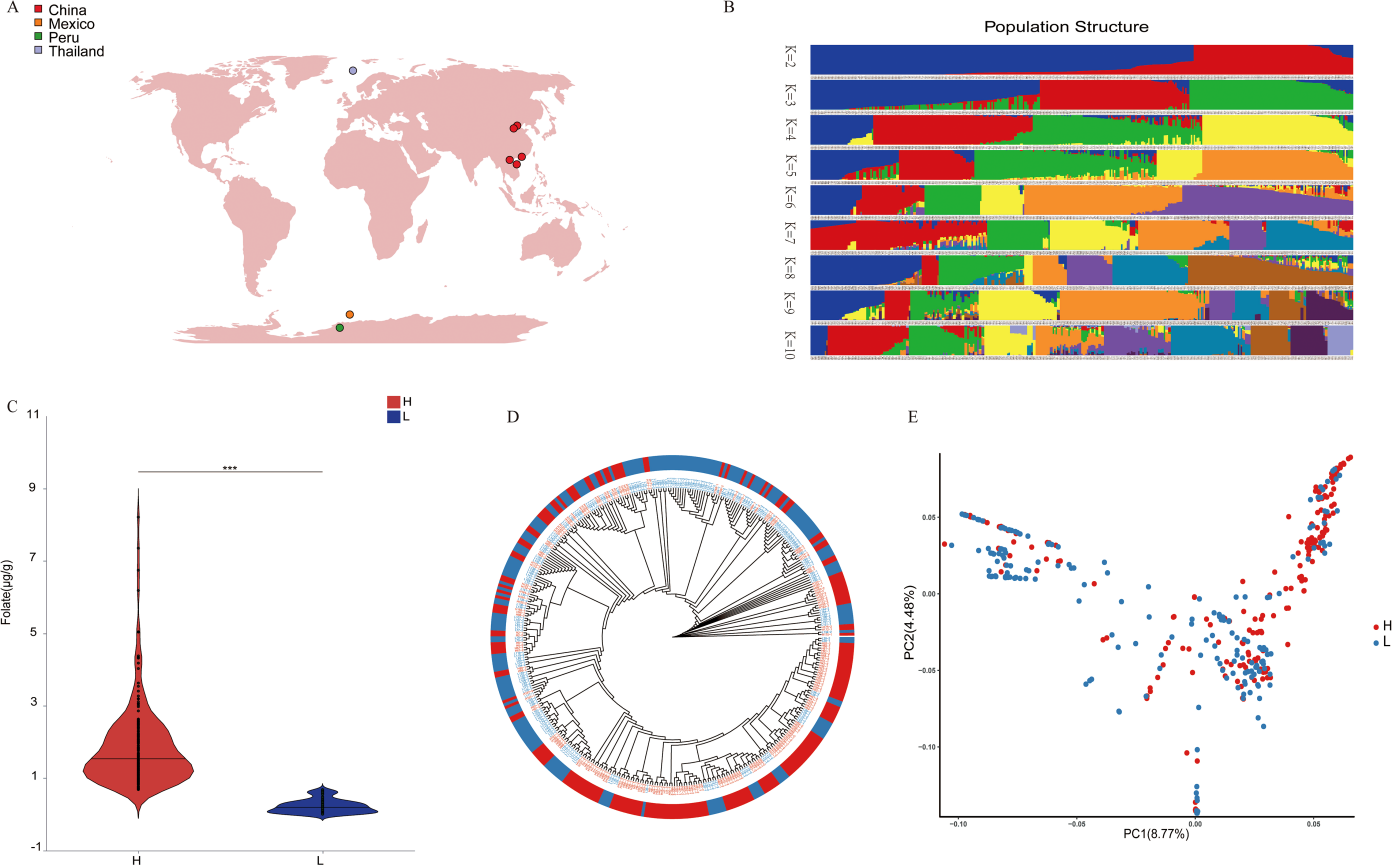
Maize genetic diversity and folate content assessment. **(A)**The geographic distribution of 380 maize accessions. **(B)** Population structure analysis results for K = 2–10. **(C)** Analysis results of folate content differences between high- and low-folate groups. **(D)** Phylogenetic neighbor-joining tree based on 380 maize samples. **(E)** PCA plot of the first two principal components for high- and low-folate groups.

To investigate the genetic basis for folate characteristics in maize, we assembled a germplasm collection comprising 380 kernel samples derived from 190 maize inbred lines representing diverse geographical origins, including China, Thailand, Mexico, and other regions (**Figure 1A**). Transcriptome sequencing analysis were conducted on a total of 380 samples. A total of 2593.07 Gb of clean data reads with a Q30 score of 94.03% were generated, with average sequencing data per sample of 5.71 Gb. Population structure analysis based on transcriptome-derived SNP markers revealed distinct genetic characteristics among four regional subpopulations (**Figure 1B**). To investigate the genetic basis of folate accumulation, we quantified kernel folate content using two biological replicates per sample. Samples were stratified into high- and low-folate groups based on median folate levels, demonstrating significant inter-group divergence in folate content (*P* < 0.01, **Figure 1C**). PCA of the folate-stratified groups showed consistent clustering patterns with phylogenetic relationships (**Figure 1D**), confirming that phenotypic variation in folate content occurred independently of genetic background (**Figure 1E**).

### Transcriptome analysis of two maize groups with difference in folate levels

**Figure 2:**
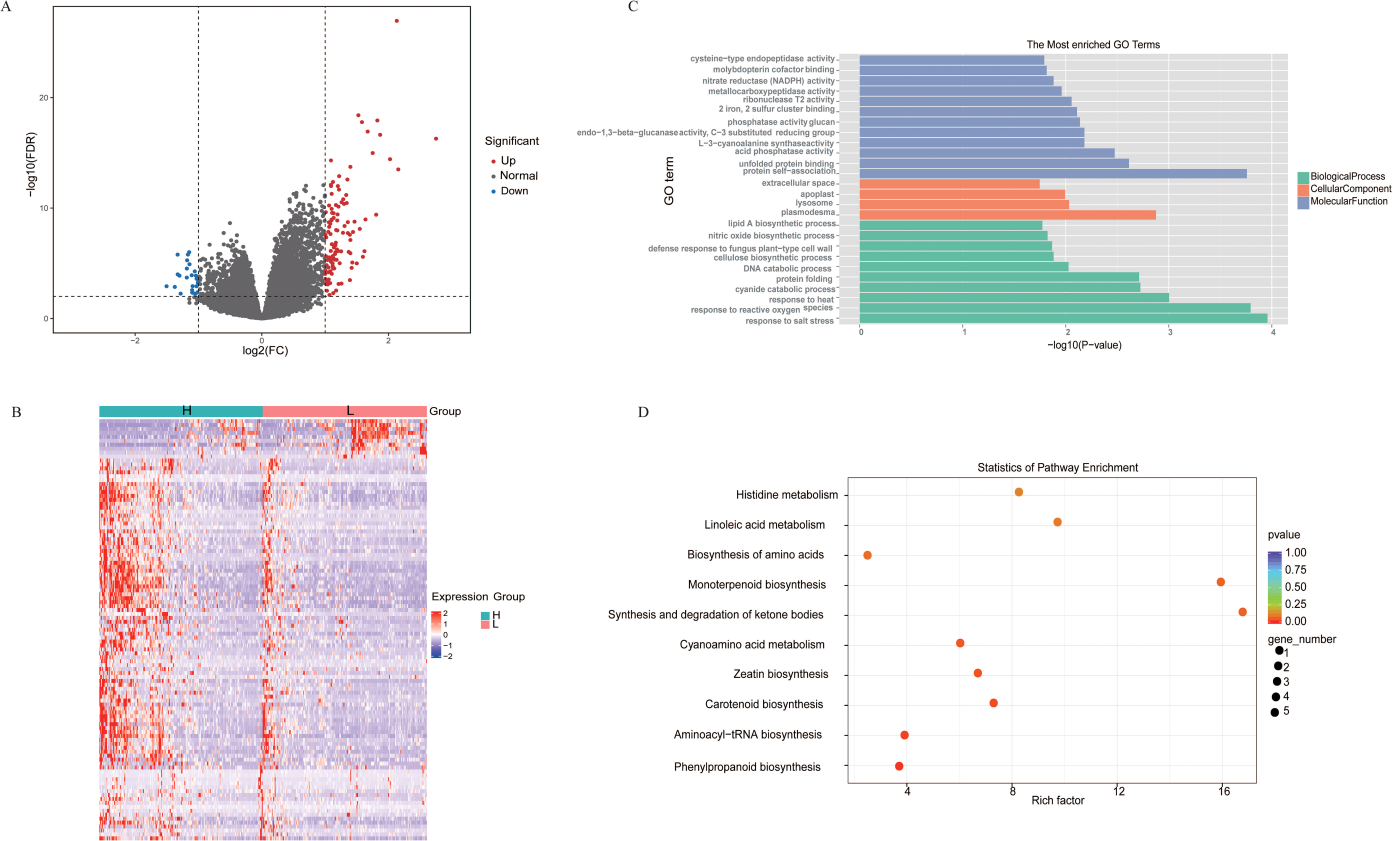
Transcriptome analysis between high- and low-folate groups. **(A)** Volcano plot of significantly DEGs between high- and low-folate groups. **(B)** Hierarchical clustering heatmap of gene expression patterns in high- and low-folate groups. **(C)** GO and **(D)** KEGG enrichment analysis of DEGs between high- and low-folate groups.

Using a threshold of Fold Change ≥ 2 and *P* value < 0.01, we identified DEGs between the high-folate and low-float groups, resulting in a total of 137 DEGs, of which 112 were upregulated and 25 were downregulated (**Figure 2A, B**). To elucidate the functional of DEGs, we performed GO enrichment analysis using a significance threshold of *P* < 0.05 across three categories: Molecular Function (MF), Biological Process (BP), and Cellular Component (CC) (**Figure 2C**). In the MF category, the top five enriched GO terms were: protein self-association, unfolded protein binding, acid phosphatase activity, L-3-cyanoalanine synthase activity, glucan endo-1,3-beta-glucanase activity, and phosphatase activity. These results suggest that the DEGs may participate in biochemical pathways associated with cyanide amino acid metabolism, phosphatase-related metabolic regulation, and protein homeostasis. In the BP category, the most significantly enriched terms included cyanide catabolic process, DNA catabolic process, protein folding, and response to reactive oxygen species (ROS). These findings indicate that the DEGs are likely involved in critical biological mechanisms such as DNA damage repair, stress responses to external stimuli, and metabolic regulation. In the CC category, the top five enriched GO terms were plasmodesma, lysosome, apoplast, extracellular space, and integral component of membrane. This implies that the DEGs may contribute to intercellular communication, substrate transport, and maintenance of cellular architecture. KEGG enrichment analysis revealed the DEGs were enriched in phenylpropanoid biosynthesis, minoacyl-tRNA biosynthesis, carotenoid biosynthesis, ceatin biosynthesis, and cyanoamino acid metabolism (**Figure 2D**).

### GWAS analysis and identification of folate-related genes

**Figure 3:**
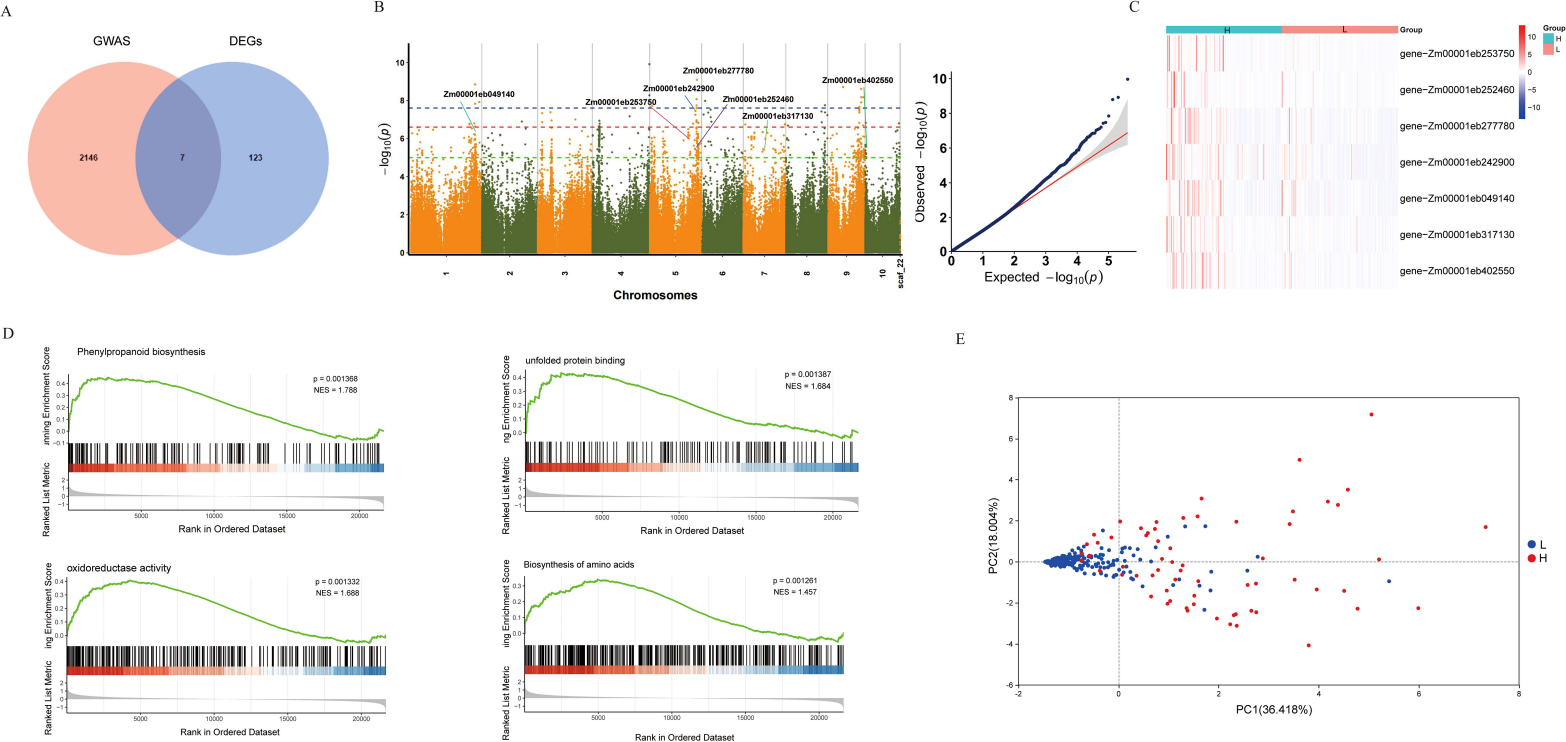
GWAS and transcriptome analysis identify folate-related candidate genes in maize. **(A)**Venn diagram intersection of GWAS and DEGs. **(B)** Manhattan and QQ plots of GWAS for folate content traits. **(C)** Hierarchical clustering heatmap of candidate genes in high- and low-folate groups. **(D)** GSEA of DEGs between high- and low-folate groups. **(E)** PCA of maize samples based on candidate gene expression profiles.

To further elucidate the regulatory mechanisms of folate expression in maize endosperm, we conducted a GWAS using the LMM method implemented in GEMMA software and analyzed the candidate gene. We employed a significance threshold adjusted by the 1% Bonferroni correction (*P*-value ≤ 1.00E-05) to identify significant associations. By integrating the Manhattan plots for folate traits and LD decay rates of 10 chromosomes in 380 maizes, and based on the LD coefficient decreasing to half of its maximum at a distance of 100 kb, we selected target intervals at 2 kb upstream and downstream of the SNP, and finally identified 692 significantly associated loci (**Figure 3B**). These loci included 3,943 SNPs associated with folate traits (**Supplementary Table S3**). Based on analysis of genes in LD regions, a total of 2,153 candidate genes were identified for potential folate characteristic associations.

### Seven candidate genes were discovered by integrating GWAS and transcriptome data

We further identified the core genes associated with folate in maize by integrating GWAS with DEGs (**Figure 3A**). They were seven genes, including *Zm00001eb252460*, *Zm00001eb049140*, *Zm00001eb242900*, *Zm00001eb277780*, *Zm00001eb402550*, *Zm00001eb317130*, *Zm00001eb253750*, were found to be located on the SNPs identified in the GWAS (**Figure 3B**), and showed significant differences in expression levels between the low and high folate groups (**Figures 3C, E**). Therefore, these genes were identified as candidate genes associated with folate accumulation. We performed GSEA to further understand the functions of the hub gene, and the results revealed that these genes are significantly enriched in several biological pathways, including biosynthesis of amino acids, phenylpropanoid biosynthesis, oxidoreductase activity, and unfolded protein binding (**Figure 3D**).

### Thirteen candidate genes regulating folate were identified by TWAS

**Figure 4:**
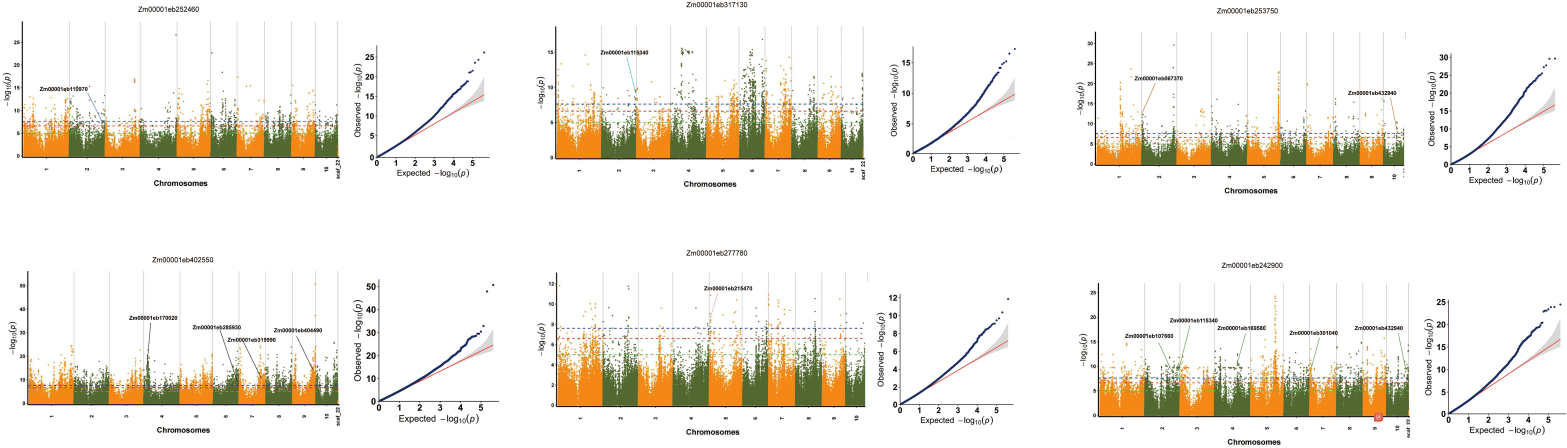
Identification of key genes regulating folate content by TWAS. The -log_10_(P) values from TWAS (y-axis) are plotted against gene positions (x-axis) on each of the chromosomes. The blue dashed line indicates the significance threshold (P < 2.476×10^-8^), with genes surpassing this threshold defined as TWAS significant genes.

To further investigate the causal genes associated with the seven core genes, we performed TWAS to identify potential genes linked to these core genes. In total, 13 candidate regulatory genes were detected with a significance threshold of *P* < 2.476e-08 (**Figure 4A**). It was worth noting that these genes identified by TWAS were significantly associated folate synthesis. *Zm00001eb112740* and *Zm00001eb169580* were annotated with dihydrofolate reductase activity and participation in the tetrahydrofolate (THF) biosynthetic process, indicating their central roles in folate metabolism by directly influencing the production of the active form of folate. The *Zm00001eb107660* gene was linked to the biosynthesis of folate-containing compounds, 5-formyltetrahydrofolate cyclo-ligase activity, and tetrahydrofolate interconversion, suggesting its regulatory role in the synthesis and metabolic transformation of folate and its bioactive derivatives. Additionally, multiple genes, including *Zm00001eb432940*, *Zm00001eb067370*, *Zm00001eb146170*, *Zm00001eb170020*, *Zm00001eb319990*, and *Zm00001eb404490*, were implicated in THF synthesis and interconversion processes. Notably, *Zm00001eb115340*, *Zm00001eb215470*, *Zm00001eb301040*, and *Zm00001eb285930* remain unannotated but represent potential candidates for folate biosynthesis, warranting further functional validation. The KEGG annotation analysis of the 13 identified genes is presented in **Figure 4B**; **Supplementary Table S4**.

### Expression verification of candidate genes

**Figure 5:**
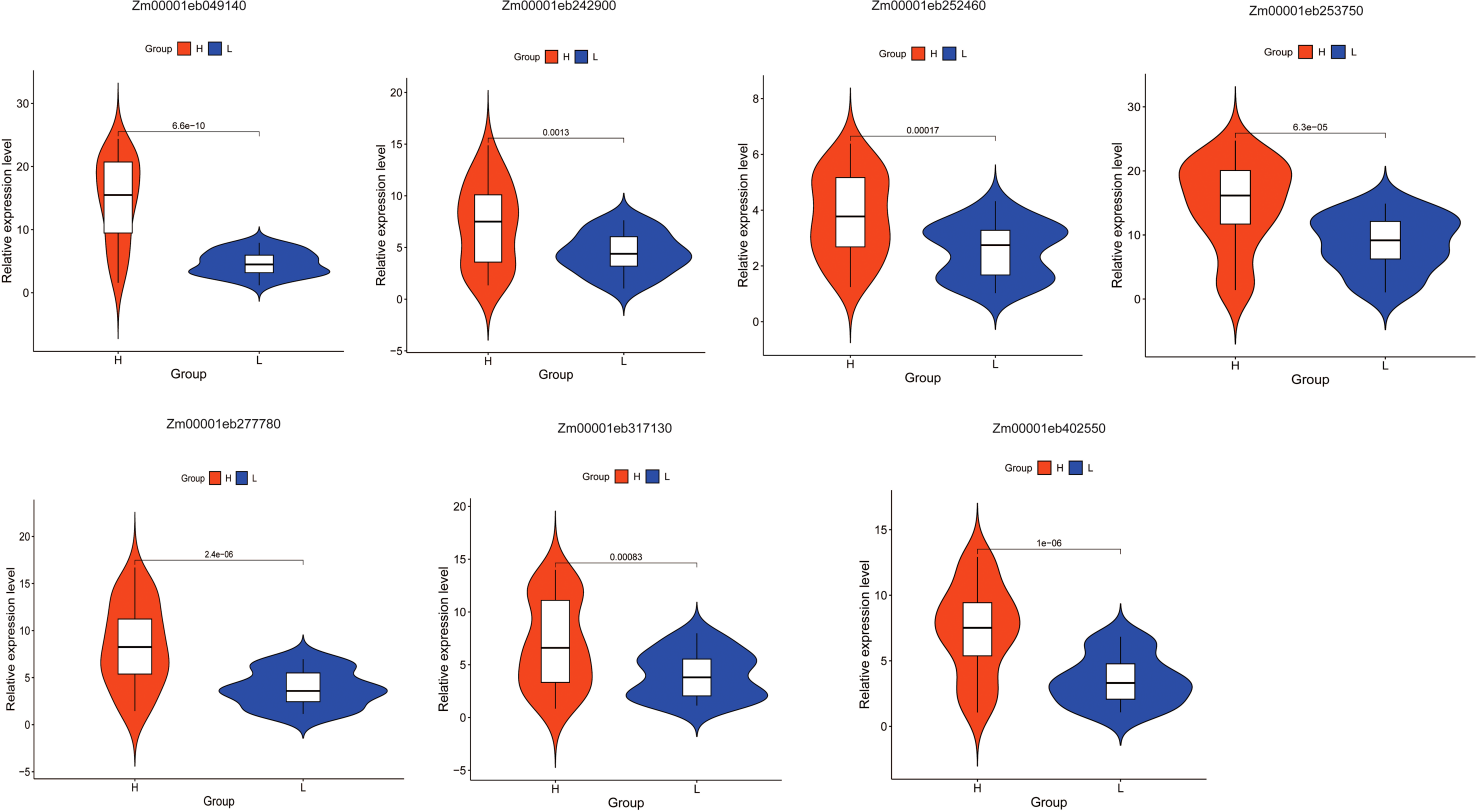
qRT-PCR validation of folate-related candidate genes.

To further analyze the correlation between these candidate genes and folate expression, we extracted RNA from 32 maize kernels each from both the high and low folate groups and performed qRT-PCR analysis to examine the expression levels of these candidate genes across the two groups. The results revealed that these seven core genes exhibited significantly higher expression levels in the high-folate group compared to the low-folate group (**Figure 5**), consistent with the transcriptomic data analysis. Furthermore, we also conducted expression analysis on 13 causal genes selected from these core genes through qRT-PCR. The results showed that 11 out of 13 genes, including *Zm00001eb107660*, *Zm00001eb112740*, *Zm00001eb115340*, *Zm00001eb146170*, Zm00001eb169580, *Zm00001eb215470*, *Zm00001eb285930*, *Zm00001eb301040*, *Zm00001eb319990*, *Zm00001eb404490*, and *Zm00001eb432940*, were significantly higher in the high folate groups compared to the low folate group (P < 0.05). The remaining two genes exhibited differences between the two groups, but these differences were not statistically significant (**Supplementary Figure S1**).

## Discussion

Maize, as one of the world’s primary cereal crops, plays a vital role in both human diets and animal feed. Known for its rich content of carbohydrates, proteins, and fats, maize also provides an abundant supply of vitamins and minerals, making it an important source of dietary folate. Despite the significant nutritional benefits of maize, its grain folate content is relatively low, which limits its potential as a source of folate supplementation and contributes to folate deficiency (Crider et al., 2011). To enhance folate levels in maize grains, it is essential to thoroughly understand the regulatory mechanisms underlying folate metabolism. With the development of sequencing technology, GWAS and TWAS have emerged as powerful tools for exploring the genetic basis of complex traits (Barbeira et al., 2018). In this study, we employed an integrated approach combining transcriptomic and genomic analyses to identify key genes and elucidate the molecular mechanisms underlying folate metabolism regulation in maize. Our findings provide a theoretical foundation for enhancing folate biosynthesis in maize grains, thereby improving their nutritional value and potential health benefits.

In this study, we conducted a transcriptome analysis comparing maize populations with high and low folate levels, identifying a total of 137 DEGs. These DEGs are primarily enriched in pathways such as phenylpropanoid biosynthesis, aminoacyl-tRNA biosynthesis, linoleic acid metabolism, zeatin biosynthesis, and cyanoamino acid metabolism. In the phenylpropanoid biosynthesis pathway, phenylalanine and trans-cinnamic acid are catalyzed by various enzymes and undergo reactions such as methylation and acylation to produce a series of phenylpropanoid compounds (Lv et al., 2024). Watanabe et al. found that phenylalanine can activate folate biosynthesis in spinach by increasing the levels of pteridine and p-aminobenzoic acid, thereby significantly enhancing folate content (Watanabe et al., 2017). Therefore, phenylalanine metabolism in the phenylpropanoid biosynthesis pathway plays a crucial role in folate synthesis. GO enrichment analysis further elucidated the roles of these DEGs in MF, CC, and BP. Notably, there is an important metabolic interaction between folate metabolism and riboflavin. In plants, riboflavin serves as the essential precursor for flavin adenine dinucleotide (FAD) biosynthesis, a vital redox cofactor that mediates electron transfer reactions in folate metabolism. FAD acts as an obligatory coenzyme for several key folate-metabolizing enzymes, which catalyzes the irreversible conversion of 5,10-methylenetetrahydrofolate to 5-methyltetrahydrofolate - a critical step in the folate cycle that influences one-carbon metabolism and methylation processes (Hanson and Gregory, 2011).These results suggest that the identified DEGs are likely involved in the regulation of folate metabolism through multiple mechanisms, including the provision of folate precursors, modulation of coenzyme availability, and participation in stress-responsive and metabolic pathways.

GWAS can associate genetic variations with complex traits, thereby efficiently identifying gene loci significantly related to plant traits (Tian et al., 2011). In our study, GWAS was employed to associate genes with folate traits, and by intersecting the differentially expressed genes between high and low folate groups, we identified seven candidate genes related to folate. Among them, *Zm00001eb277780*, located on chromosome 6 in maize, encodes a protein involved in FAD_binding and oxidoreductase activity. FAD is a crucial redox cofactor in various enzyme-catalyzed oxidation-reduction reactions, essential for numerous enzymes in folate metabolism (Roje, 2007). *Zm00001eb252460* is implicated in protein binding functions, and research suggests that many enzymes in folate metabolism require proper folding and stability to function correctly (Zheng and Cantley, 2019). This gene may impact folate metabolism by maintaining the folding and stability of folate metabolic enzymes. These insights highlight the potential of GWAS in uncovering complex genetic networks and offer new avenues for enhancing folate content in crops through targeted genetic interventions.

TWAS can provide single gene resolution for candidate genes in plants, complementing GWAS (Li et al., 2024). To delve deeper into the regulatory mechanisms governing folate expression, this study analyzed genes at genetic loci significantly associated with seven core genes, ultimately identifying 13 pivotal genes by TWAS analysis. Among these, *Zm00001eb112740* and *Zm00001eb169580* were annotated with DHFR activity and involvement in the THF biosynthetic process, positioning them as central players in folate metabolism. DHFR catalyzes the reduction of DHF to THF, the active coenzyme forms essential for one-carbon transfer reactions in nucleotide synthesis and methylation processes (Jabrin et al., 2003). These results corroborate prior research indicating the essential role of DHFR activity in sustaining folate bioavailability in plants, with supporting evidence from folate-biofortified rice cultivars (Liang et al., 2024).The *Zm00001eb107660* gene, associated with folate-containing compound biosynthesis, 5-formyltetrahydrofolate cyclo-ligase activity, and THF interconversion, suggests a regulatory role in modulating folate derivatives. This enzyme likely facilitates the conversion of 5-formyl-THF to other THF forms, a process critical for maintaining folate pool plasticity under varying metabolic demands (Li et al., 2021). The functional genomic investigation has identified a cohort of key regulatory genes, including *Zm00001eb432940*, *Zm00001eb067370*, *Zm00001eb146170*, *Zm00001eb170020*, *Zm00001eb319990*, and *Zm00001eb404490*, that are critically involved in THF biosynthesis and interconversion pathways. These genes appear to coordinately regulate THF synthesis, derivative conversion, and metabolic flux partitioning, thereby playing pivotal roles in maintaining folate homeostasis in maize. Notably, the identification of unannotated genes (*Zm00001eb115340*, *Zm00001eb301040*, and *Zm00001eb285930*) suggests the existence of previously unrecognized functional modules within the folate metabolic network. Elucidation of their biological functions may uncover novel regulatory mechanisms and provide new research directions for folate metabolic engineering in maize. Notably, the folate-related candidate genes identified through GWAS in this study show no significant homology to known folate metabolism regulatory genes in Arabidopsis and rice. These findings suggest that these candidate genes may represent novel components of folate metabolism regulation in maize, with functional mechanisms distinct from those of reported folate-related genes in model plants. These results delineate maize-specific regulatory genes of folate biosynthesis. Functional characterization of these genes may further reveal novel mechanisms controlling their accumulation in maize. In summary, these genes are indispensable in the metabolism and regulation of folate, providing an essential molecular foundation for the deeper understanding of folate expression regulatory mechanisms. These insights are crucial for developing strategies to enhance folate content in crops, with potential implications for nutritional improvement and agricultural productivity.

## Conclusion

This study presents the first large-scale investigation combining GWAS with TWAS to systematically identify candidate genes associated with folate accumulation in maize. The expression level of these genes was experimentally validated using qRT-PCR and functional assays, confirming their roles in folate biosynthesis and regulation. Our findings not only advance the molecular understanding of folate accumulation in maize but also identify promising genetic targets for biofortification strategies, offering a foundation for enhancing nutritional quality in maize through precision breeding and biotechnological applications. A limitation of this study is the lack of functional validation through genetic approaches such as gene knockout or overexpression. Future investigations should employ these methods to further elucidate the underlying molecular mechanisms.

## Data Availability

The data presented in the study are deposited in the NCBI Sequence Read Archive (SRA) repository, accession number PRJNA12623333.

## Supplementary material

Supplementary Table S1 The detailed information of 380 maize grain samples.

Supplementary Table S2 The detailed information of qRT-PCR primers.

Supplementary Table S3 SNPs associated with folate traits identified by GWAS.

Supplementary Table S4 The functional annotation results of candidate genes.

Supplementary Figure S1 qRT-PCR validation of folate-related candidate genes identified by TWAS.

## References

[B1] Alexander D. H. Novembre J. Lange K. (2009). Fast model-based estimation of ancestry in unrelated individuals. Genome Res. 19, 1655–1664. doi: 10.1101/gr.094052.109 19648217 PMC2752134

[B2] Barbeira A. N. Dickinson S. P. Bonazzola R. Zheng J. Wheeler H. E. Torres J. M.. (2018). Exploring the phenotypic consequences of tissue specific gene expression variation inferred from GWAS summary statistics. Nat. Commun. 9, 1825. doi: 10.1038/s41467-018-03621-1 29739930 PMC5940825

[B3] Blancquaert D. Van Daele J. Strobbe S. Kiekens F. Storozhenko S. De Steur H.. (2015). Improving folate (vitamin B9) stability in biofortified rice through metabolic engineering. Nat. Biotechnol. 33, 1076–1078. doi: 10.1038/nbt.3358 26389575

[B4] Crider K. S. Bailey L. B. Berry R. J. (2011). Folic acid food fortification-its history, effect, concerns, and future directions. Nutrients. 3, 370–384. doi: 10.3390/nu3030370 22254102 PMC3257747

[B5] Guo W. Lian T. Wang B. Guan J. Yuan D. Wang H.. (2019). Genetic mapping of folate QTLs using a segregated population in maize. J. Integr. Plant Biol. 61, 675–690. doi: 10.1111/jipb.12811 30938052

[B6] Gusev A. Ko A. Shi H. Bhatia G. Chung W. Penninx B. W.. (2016). Integrative approaches for large-scale transcriptome-wide association studies. Nat. Genet. 48, 245–252. doi: 10.1038/ng.3506 26854917 PMC4767558

[B7] Hanson A. D. Gregory J. F. 3rd (2011). Folate biosynthesis, turnover, and transport in plants. Annu. Rev. Plant Biol. 62, 105–125. doi: 10.1146/annurev-arplant-042110-103819 21275646

[B8] Hou S. Men Y. Zhang Y. Zhao K. Ma G. Li H.. (2022). Role of miRNAs in regulation of SA-mediated upregulation of genes involved in folate and methionine metabolism in foxtail millet. Front. Plant Sci. 13, 1023764. doi: 10.3389/fpls.2022.1023764 36561440 PMC9763449

[B9] Jabrin S. Ravanel S. Gambonnet B. Douce R. Rébeillé F. (2003). One-carbon metabolism in plants. Regulation of tetrahydrofolate synthesis during germination and seedling development. Plant Physiol. 131, 1431–1439. doi: 10.1104/pp.016915 12644692 PMC166902

[B10] Kumar S. Stecher G. Li M. Knyaz C. Tamura K. (2018). MEGA X: molecular evolutionary genetics analysis across computing platforms. Mol. Biol. Evol. 35, 1547–1549. doi: 10.1093/molbev/msy096 29722887 PMC5967553

[B11] Li W. Liang Q. Mishra R. C. Sanchez-Mu Oz R. Wang H. Chen X.. (2021). The 5-formyl-tetrahydrofolate proteome links folates with C/N metabolism and reveals feedback regulation of folate biosynthesis. Plant Cell. 33, 3367–3385. doi: 10.1093/plcell/koab198 34352110 PMC8505879

[B12] Li D. Wang Q. Tian Y. Lyv X. Zhang H. Hong H.. (2024). TWAS facilitates gene-scale trait genetic dissection through gene expression, structural variations, and alternative splicing in soybean. Plant Commun. 5, 101010. doi: 10.1016/j.xplc.2024.101010 38918950 PMC11573905

[B13] Lian T. Wang X. Li S. Jiang H. Zhang C. Wang H.. (2022). Comparative transcriptome analysis reveals mechanisms of folate accumulation in maize grains. Int. J. Mol. Sci. 23, 1708. doi: 10.3390/ijms23031708 35163628 PMC8836222

[B14] Liang Q. Zhang W. Pang J. Zhang S. Hou X. Zhang C. (2024). Creation of folate-biofortified rice by simultaneously enhancing biosynthetic flux and blocking folate oxidation. Mol. Plant 17, 1487–1489. doi: 10.1016/j.molp.2024.09.005 39289873

[B15] Lv M. Zhang L. Wang Y. Ma L. Yang Y. Zhou X.. (2024). Floral volatile benzenoids/phenylpropanoids: biosynthetic pathway, regulation and ecological value. Hortic. Res. 11, uhae220. doi: 10.1093/hr/uhae220 39398951 PMC11469922

[B16] Pan Q. Jia R. Pi K. Tang Q. Zhang J. Huang Y.. (2024). Revealing the key role of the folate synthesis regulatory gene DHNA in tobacco leaf yellowing based on BSA-seq, RNA-seq, and proteomic sequencing. BMC Plant Biol. 24, 1211. doi: 10.1186/s12870-024-05917-5 39701982 PMC11656980

[B17] Price A. L. Patterson N. J. Plenge R. M. Weinblatt M. E. Shadick N. A. Reich D. (2006). Principal components analysis corrects for stratification in genome-wide association studies. Nat. Genet. 38, 904–909. doi: 10.1038/ng1847 16862161

[B18] Roje S. (2007). Vitamin B biosynthesis in plants. Phytochemistry. 68, 1904–1921. doi: 10.1016/j.phytochem.2007.03.038 17512961

[B19] Shulpekova Y. Nechaev V. Kardasheva S. Sedova A. Kurbatova A. Bueverova E.. (2021). The Concept of Folic Acid in Health and Disease. Molecules 26, 3731. doi: 10.3390/molecules26123731 34207319 PMC8235569

[B20] Song L. Yu D. Zheng H. Wu G. Sun Y. Li P.. (2021). Weighted gene co-expression network analysis unveils gene networks regulating folate biosynthesis in maize endosperm. 3 Biotech. 11, 441. doi: 10.1007/s13205-021-02974-7 PMC8455765 34631342

[B21] Su N. Li J. Xia Y. Huang C. Chen L. (2024). Non-causal relationship of polycystic ovarian syndrome with homocysteine and B vitamins: evidence from a two-sample Mendelian randomization. Front. Endocrinol. (Lausanne). 15, 1393847. doi: 10.3389/fendo.2024.1393847 38841299 PMC11150916

[B22] Tian F. Bradbury P. J. Brown P. J. Hung H. Sun Q. Flint-Garcia S.. (2011). Genome-wide association study of leaf architecture in the maize nested association mapping population. Nat. Genet. 43, 159–162. doi: 10.1038/ng.746 21217756

[B23] Visscher P. M. Wray N. R. Zhang Q. Sklar P. McCarthy M. I. Brown M. A.. (2017). 10 years of GWAS discovery: biology, function, and translation. Am. J. Hum. Genet. 101, 5–22. doi: 10.1016/j.ajhg.2017.06.005 28686856 PMC5501872

[B24] Wainberg M. Sinnott-Armstrong N. Mancuso N. Barbeira A. N. Knowles D. A. Golan D.. (2019). Opportunities and challenges for transcriptome-wide association studies. Nat. Genet. 51, 592–599. doi: 10.1038/s41588-019-0385-z 30926968 PMC6777347

[B25] Watanabe S. Ohtani Y. Tatsukami Y. Aoki W. Amemiya T. Sukekiyo Y.. (2017). Folate biofortification in hydroponically cultivated spinach by the addition of phenylalanine. J. Agric. Food Chem. 65, 4605–4610. doi: 10.1021/acs.jafc.7b01375 28548831

[B26] Xia H. Zhang Z. Luo C. Wei K. Li X. Mu X.. (2023). MultiPrime: A reliable and efficient tool for targeted next-generation sequencing. iMeta. 2, e143. doi: 10.1002/imt2.v2.4 38868227 PMC10989836

[B27] Xiao Y. Yu Y. Xie L. Li K. Guo X. Li G.. (2022). A genome-wide association study of folates in sweet corn kernels. Front. Plant Sci. 13, 1004455. doi: 10.3389/fpls.2022.1004455 36247547 PMC9562826

[B28] Young M. D. Wakefield M. J. Smyth G. K. Oshlack A. (2010). Gene ontology analysis for RNA-seq: accounting for selection bias. Genome Biol. 11, R14. doi: 10.1186/gb-2010-11-2-r14 20132535 PMC2872874

[B29] Yu G. Wang L. G. Han Y. He Q. Y. (2012). clusterProfiler: an R package for comparing biological themes among gene clusters. Omics. 16, 284–287. doi: 10.1089/omi.2011.0118 22455463 PMC3339379

[B30] Yuan X. Jiang X. Zhang M. Wang L. Jiao W. Chen H.. (2024). Integrative omics analysis elucidates the genetic basis underlying seed weight and oil content in soybean. Plant Cell. 36, 2160–2175. doi: 10.1093/plcell/koae062 38412459 PMC11132872

[B31] Zheng Y. Cantley L. C. (2019). Toward a better understanding of folate metabolism in health and disease. J. Exp. Med. 216, 253–266. doi: 10.1084/jem.20181965 30587505 PMC6363433

[B32] Zhou X. Stephens M. (2012). Genome-wide efficient mixed-model analysis for association studies. Nat. Genet. 44, 821–824. doi: 10.1038/ng.2310 22706312 PMC3386377

