# Supplementary figures and images for: Genome and transcriptome wide association study identify candidate genes regulating folate levels in maize
Source: Front Plant Sci. 2025 Jun 19;16:1606220. doi: 10.3389/fpls.2025.1606220 (PMC12222111; doi:10.3389/fpls.2025.1606220)

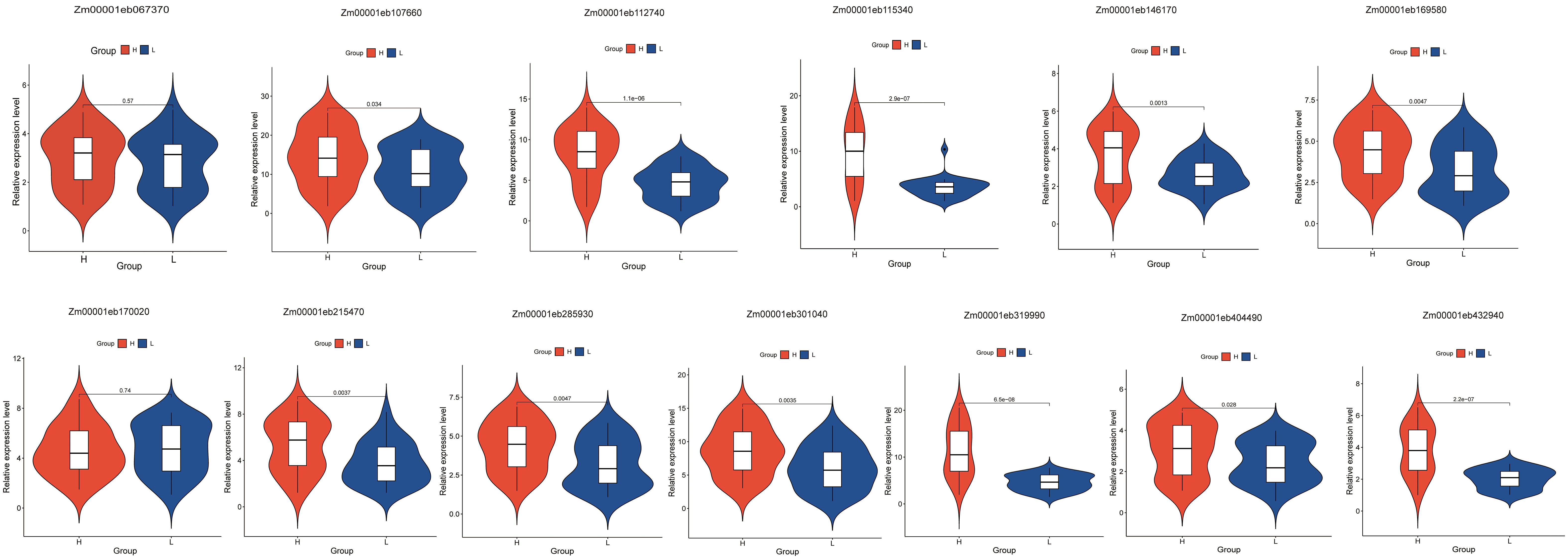

Supplement: Supplementary Figure S1 — qRT-PCR validation of folate-related candidate genes identified by TWAS. [file Image1.jpeg]
